# Understanding the relationship between cuproptosis and the development of hepatocellular carcinoma: implications for targeted therapies

**DOI:** 10.3389/fimmu.2025.1557223

**Published:** 2025-03-12

**Authors:** Haixia Zhu, Yamin Zhao, Yaxuan Wang, Guohua Wei, Jibin Liu

**Affiliations:** ^1^ Institute of Oncology, Affiliated Tumor Hospital of Nantong University & Nantong Tumor Hospital, Nantong, China; ^2^ Internal Medicine, The Second People’s Hospital of Nantong, Nantong, China

**Keywords:** hepatocellular carcinoma, cuproptosis, prognosis, treatment resistance, immune microenvironment

## Abstract

Hepatocellular carcinoma (HCC) presents a significant global health challenge, particularly in developing countries where its incidence is markedly elevated. Despite advancements in treatment modalities, the high malignancy, metastatic potential, and drug resistance associated with HCC contribute to poor clinical outcomes, underscoring the necessity for a more profound understanding of its pathogenesis. This review meticulously examines the role of copper apoptosis, a novel form of programmed cell death linked to dysregulated copper metabolism, in the development and progression of HCC. By conducting a comprehensive review of recent literature and experimental studies, we elucidate the molecular mechanisms through which excess copper induces oxidative stress, pyroptosis, and ferroptosis, thereby influencing tumorigenesis and progression. This review offers valuable insights into the intricate relationship between copper metabolism and HCC, positioning copper apoptosis as a potential therapeutic target to enhance treatment strategies and improve patient outcomes.

## 1 Introduction

The incidence of hepatocellular carcinoma (HCC) continues to rise globally, particularly in developing countries (1). This phenomenon has attracted considerable interest because of liver cancer’s effect on patients’ quality of life and the considerable difficulties it poses to public health systems. The occurrence of liver cancer varies significantly across various regions; epidemiological research shows that the rates in Asia and Africa are notably elevated compared to those in North America and Europe (2). This discrepancy is primarily linked to the prevalence of hepatitis virus infections and liver cirrhosis in specific areas. In my country, liver cancer ranks fourth in incidence among all malignant tumors and second in mortality attributed to cancer (3). HCC, the most prevalent form of primary liver cancer in my country, often develops from cirrhosis and chronic hepatitis, with key risk factors including hepatitis B and C virus infections, long-term alcohol consumption, obesity, and metabolic syndrome (4). The incidence and mortality rates of HCC remain alarmingly high and continue to escalate annually, posing a serious threat to public health (5). Existing therapeutic approaches for HCC encompass surgical resection, ablation techniques, transhepatic arterial chemoembolization, targeted therapies, immunological treatments, and chemoradiation. However, the substantial malignancy, propensity for metastasis, and resistance to pharmacological interventions associated with HCC result in less than optimal clinical outcomes (6). Therefore, a comprehensive understanding of the pathogenesis of HCC is crucial for enhancing therapeutic efficacy. Copper is an essential trace element for the human body, playing important auxiliary roles in various cellular processes, including mitochondrial respiration, antioxidant defense, and the synthesis of biological complexes (7). It is also involved in the activity of numerous enzymes and biological functions. However, the same properties that make copper essential can lead to toxicity when it accumulates excessively, potentially causing increased intracellular oxidative stress and disrupting cellular function (8). The liver plays a crucial role in copper metabolism by regulating various biological processes and producing and releasing copper-binding proteins. Disruptions in copper metabolism can adversely affect liver function. Recent studies on the regulatory mechanisms of cell death, particularly the identification of cuproptosis—a novel form of programmed cell death—offer new insights for the research and treatment of hepatocellular carcinoma HCC (9). In HCC, dysregulated copper metabolism is closely linked to tumor development, with abnormal copper ion levels directly or indirectly influencing the formation, proliferation, and metastasis of HCC (10). Consequently, the role of cuproptosis in HCC has emerged as a critical area for investigation. This review aims to systematically summarize and analyze the mechanisms of cuproptosis in the occurrence and progression of HCC, as well as the advancements in its diagnosis and treatment.

## 2 Concept and molecular mechanism of cuproptosis

**Figure 1:**
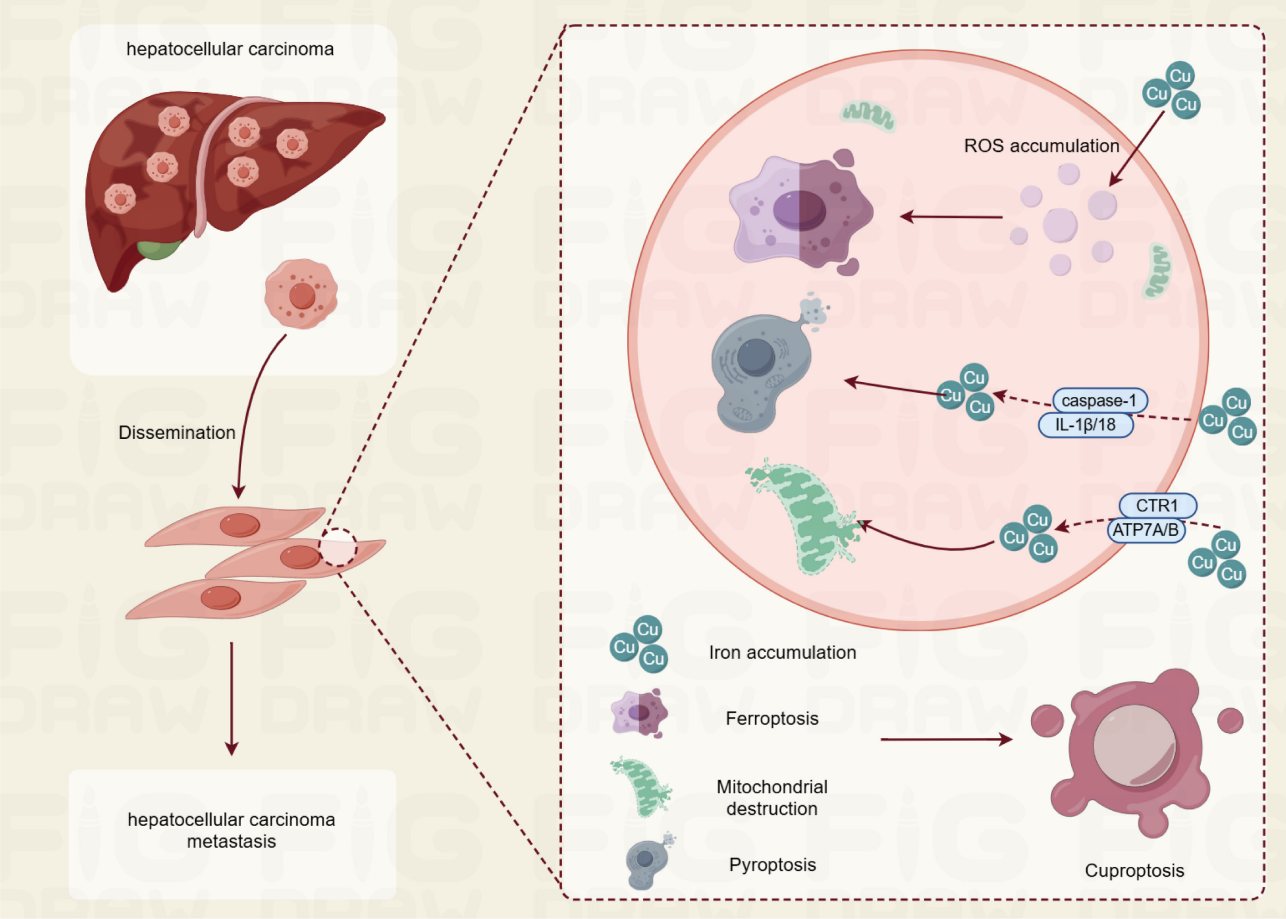
The role of copper apoptosis in hepatocellular carcinoma pathogenesis.

The term “cuproptosis” has emerged in recent years alongside a deeper understanding of copper biology. This term specifically refers to a mode of cell death induced by abnormally elevated concentrations of copper ions within cells, characterized by the depletion of intracellular copper and abnormal accumulation. As early as the late 20th century, scientists began to observe that excess copper ions could induce cytotoxic effects. However, research at that time primarily focused on the impact of copper ions on the redox balance within cells, suggesting that they could cause cell damage by generating excess reactive oxygen species (ROS). This perspective, however, does not fully elucidate the mechanisms by which copper ions specifically induce cell death. Subsequent investigations have revealed that copper ions interact with specific proteins, such as the copper transport proteins CTR1 and ATP7A/B, which are crucial for regulating the transport and distribution of copper ions within cells. These findings indicate that excess copper ions can impair mitochondrial function, disrupt energy metabolism, and ultimately prevent cells from maintaining a normal metabolic state, leading to cell death. Cuproptosis mainly plays a role through oxidative stress, pyroptosis, and ferroptosis in cells (**Figure 1**).

### 2.1 Cuproptosis and oxidative stress

Excess copper in cells can induce oxidative stress by generating ROS, which are harmful byproducts of cellular metabolism. This oxidative stress arises from the redox cycling of copper ions, particularly the transition between Cu(I) and Cu (II) states, which can catalyze the formation of ROS in the presence of oxygen and reducing agents such as ascorbate and glutathione. The accumulation of ROS can lead to lipid peroxidation, protein damage, and ultimately cell death through apoptosis or necrosis (11) (12). Recent studies have highlighted that cuproptosis is distinct from traditional apoptosis mechanisms. Instead, it is characterized by the aggregation of lipoylated proteins within the tricarboxylic acid (TCA) cycle, leading to proteotoxic stress. This aggregation disrupts normal cellular functions and contributes to cell death, suggesting that copper’s toxicity may not solely rely on oxidative stress but also on its ability to interfere with protein homeostasis (13) (14). In research models like those utilizing mice, elevated amounts of copper have demonstrated an ability to raise ROS levels while reducing the function of antioxidant enzymes, thereby highlighting a distinct relationship between copper exposure and oxidative stress. This oxidative stress correlates with impaired mitochondrial function and the triggering of apoptotic pathways, which encompass the release of cytochrome c and the activation of caspases—key mediators in the process of apoptosis (15). Moreover, the relationship between copper and oxidative stress is complex, as both excess and deficiency of copper can lead to oxidative damage. While excess copper catalyzes the production of ROS, copper deficiency impairs the function of copper-dependent antioxidant enzymes, further exacerbating oxidative stress (16) (17). This duality underscores the importance of maintaining copper homeostasis for cellular health.

### 2.2 Cuproptosis and pyroptosis

Copper accumulation is linked to pyroptosis, a pro-inflammatory form of programmed cell death. Pyroptosis is marked by the activation of caspase-1, resulting in the release of pro-inflammatory cytokines such as IL-1β and IL-18, and is frequently initiated by the activation of the inflammasome in response to cellular stress or infection. Research has demonstrated that excessive copper can induce pyroptosis through various mechanisms. For instance, in primary chicken hepatocytes exposed to elevated concentrations of copper sulfate, the expression of genes associated with pyroptosis significantly increased, including caspase-1, IL-1β, and IL-18. This indicates that copper can activate the pyroptosis pathway by generating ROS, which are known to play a crucial role in cell signaling and stress responses. Inhibition of caspase-1 has been shown to mitigate the cytotoxic effects of copper, suggesting that the activation of this protease is a pivotal event in copper-induced pyroptosis (18). Furthermore, research investigating the impact of copper on microglia demonstrated that exposure to copper triggers the NF-κB signaling pathway, a mechanism associated with inflammatory responses. This activation is associated with elevated reactive oxygen species (ROS) levels, which eventually contribute to the pyroptosis of dopaminergic neurons. The persistent buildup of copper within these cells leads to mitochondrial impairment and the increased expression of proteins related to the NLRP3/caspase-1/GSDMD pathway, thereby reinforcing copper’s involvement in facilitating pyroptosis via inflammatory mechanisms (19). Moreover, interactions between copper and other metals, such as molybdenum and cadmium, have been investigated in the context of neurotoxicity. Studies indicate that simultaneous exposure to these metals can exacerbate pyroptosis by inhibiting Nrf2-mediated antioxidant defense responses, which are essential for maintaining cellular redox balance and preventing oxidative stress-induced cell death (20).

### 2.3 Cuproptosis and ferroptosis

Copper ions play a significant role in iron metabolism, particularly in the context of ferroptosis, a form of regulated cell death characterized by iron-dependent lipid peroxidation. Recent studies have highlighted the intricate relationship between copper and iron, suggesting that copper can influence iron homeostasis and promote ferroptosis through various mechanisms. One of the key findings is that copper levels can increase in tissues during iron deficiency, indicating a reciprocal relationship between these two essential minerals. This interaction is crucial because both copper and iron are involved in redox chemistry, where they can participate in oxidation-reduction reactions that are vital for cellular functions. For instance, copper is known to act as a cofactor for enzymes that facilitate iron metabolism, thereby influencing the availability of iron in biological systems (21) (22). Moreover, copper ions can enhance the redox reactions of iron, which is particularly relevant in the context of ferroptosis. Ferroptosis is triggered by the accumulation of lipid peroxides, and iron plays a central role in this process by catalyzing the formation of reactive oxygen species (ROS) through Fenton chemistry. Copper can exacerbate this effect by promoting the oxidation of iron, thereby increasing the availability of ferrous iron (Fe²^+^) that is more reactive and capable of generating ROS (23)24]. This mechanism suggests that elevated copper levels could sensitize cells to ferroptosis, particularly in conditions where iron is also abundant. In addition to its role in enhancing iron redox reactions, copper’s involvement in the regulation of proteins associated with ferroptosis has been noted. For example, copper can influence the expression and activity of proteins that are critical for maintaining iron homeostasis, such as ceruloplasmin, which oxidizes ferrous iron to ferric iron (Fe³^+^) for transport and storage (24) (25). This regulation is essential for preventing iron overload and associated oxidative stress, which can lead to cell death. Furthermore, the interplay between copper and iron is not only limited to promoting ferroptosis but also extends to the broader context of cancer biology. Cancer cells often exhibit altered metal ion metabolism, including increased demand for copper and iron, which supports their rapid proliferation and survival.

## 3 The role of cuproptosis in HCC

A growing body of research has linked imbalances in copper metabolism to the onset and development of several diseases, with a particular emphasis on cancer. Studies indicate that serum levels of copper ions are markedly higher in individuals diagnosed with HCC, which could be closely tied to both the aggressiveness of the cancer and its prognosis. At the cellular level, an excess of copper ions has been shown to activate a variety of intracellular signaling pathways, which consequently fosters the proliferation, migration, and invasion of cancer cells. Additionally, copper plays a role in upregulating the expression of vascular endothelial growth factor (VEGF), which promotes angiogenesis in tumors. In environments with elevated copper levels, the functionality of copper-dependent enzymes may also be modified, affecting DNA repair processes within cells and subsequently heightening the risk of genetic mutations while accelerating the advancement of tumors.

### 3.1 Cuproptosis and the development of HCC

In liver cancer, the relationship between copper levels and cell proliferation is significant. Elevated copper concentrations have been associated with increased cell growth, migration, and invasion of liver cancer cells. This is partly due to the modulation of key oncogenes such as MYC, which regulates cellular growth and is often overexpressed in HCC. Studies have shown that high extracellular copper levels can sensitize liver cells to transformation, suggesting that copper may play a role in the progression from non-alcoholic fatty liver disease (NAFLD) and cirrhosis to HCC (26). The mechanism of cuproptosis involves the binding of copper to fatty acylated proteins within the tricarboxylic acid (TCA) cycle, leading to the aggregation of these proteins and subsequent proteotoxic stress. This process results in the downregulation of iron-sulfur (Fe-S) cluster proteins, which are crucial for various cellular functions, including mitochondrial respiration. The loss of these proteins contributes to the toxic effects of copper overload, ultimately leading to cell death (27) (28). Research has identified specific long non-coding RNAs (lncRNAs) that are associated with cuproptosis and may influence the proliferation of liver cancer cells. For instance, a study constructed a prognostic signature based on cuproptosis-related lncRNAs, which were found to correlate with the immune microenvironment and therapeutic responses in HCC patients. This signature was able to stratify patients into high-risk and low-risk groups based on their likelihood of survival, indicating that cuproptosis-related pathways could be leveraged for prognostic assessments and therapeutic strategies (29) (30). Moreover, the expression of copper transporters, such as SLC31A1 (which imports copper) and ATP7A (which exports excess copper), is altered in HCC. In particular, ATP7A expression is often upregulated in cancerous tissues, suggesting that liver cancer cells may adapt to high copper levels by enhancing their copper uptake and export mechanisms. This adaptation can lead to a resistance to copper-induced toxicity, allowing cancer cells to proliferate despite the presence of potentially lethal copper concentrations (31). The interplay between copper homeostasis and liver cancer proliferation highlights the potential for targeting copper metabolism as a therapeutic strategy. For example, copper chelators and ionophores have been explored as potential anticancer agents, although their clinical efficacy has been variable. The identification of biomarkers related to cuproptosis could enhance the selection of patients who might benefit from such treatments, particularly those with a metabolic profile characterized by high levels of lipoylated TCA cycle enzymes, which are more sensitive to cuproptosis. In summary, cuproptosis plays a critical role in the proliferation of liver cancer cells by influencing cellular metabolism and survival pathways.

### 3.2 Cuproptosis and prognosis of HCC

The role of cuproptosis in HCC is still being explored, but preliminary studies suggest that it may have important implications for prognosis and treatment strategies. Recent investigations have identified specific long noncoding RNAs (lncRNAs) associated with cuproptosis that could serve as prognostic markers for HCC. For instance, a study utilizing The Cancer Genome Atlas (TCGA) dataset developed a prognostic signature based on cuproptosis-related lncRNAs, which included four key lncRNAs: AL5907053, LINC02870, KDM4A-AS1, and MKLN1-AS. These lncRNAs were shown to correlate with patient survival outcomes, allowing for the classification of HCC patients into high-risk and low-risk groups based on their expression levels. The prognostic model demonstrated significant predictive power, with area under the curve (AUC) values for 1-, 3-, and 5-year survival rates indicating its effectiveness in stratifying patient risk. Moreover, the study highlighted that the high-risk group exhibited distinct immune profiles and differences in drug sensitivity, suggesting that cuproptosis-related lncRNAs not only influence tumor progression but also interact with the tumor immune microenvironment. This interaction may provide insights into the efficacy of immunotherapy in HCC patients, as certain immune checkpoint genes and immune cell subpopulations were found to differ significantly between the risk groups (29). The link between copper regulation and the advancement of cancer is highlighted by the fact that copper concentrations frequently increase in HCC tissues. This build-up can facilitate tumor development and spread, positioning copper as a possible target for therapy. The investigation into copper chelators and ionophores as potential treatment alternatives is gaining attention, as these agents might suppress tumor growth by triggering cuproptosis in cancerous cells. For instance, copper ionophores have shown promise in preclinical studies, suggesting that manipulating copper levels could be a viable strategy for HCC treatment (32). In addition to the prognostic implications, understanding the mechanisms of cuproptosis could lead to the identification of new therapeutic targets. The involvement of specific genes, such as FDX1, which plays a crucial role in cuproptosis, highlights the potential for developing targeted therapies that exploit the vulnerabilities of HCC cells to copper dysregulation.

### 3.3 Cuproptosis and the immune microenvironment of HCC

In HCC, the immune microenvironment plays a crucial role in tumor development and progression. The tumor microenvironment (TME) is composed of various cell types, including immune cells, stromal cells, and extracellular matrix components, which interact dynamically to influence tumor behavior. Recent studies have shown that cuproptosis is associated with changes in immune cell infiltration within the TME. For instance, different subtypes of cuproptosis regulation in HCC have been identified, revealing distinct immune cell infiltration patterns that correlate with patient prognosis (33). High-risk groups, characterized by elevated cuproptosis-related gene expression, exhibit greater immune and stromal cell infiltration, which is often associated with a poorer prognosis (34). Moreover, the relationship between cuproptosis and immune checkpoint pathways has been explored. The expression of immune checkpoint molecules, such as PD-L1, varies significantly between high- and low-risk groups of HCC patients, suggesting that cuproptosis may influence the efficacy of immunotherapy (35). The presence of immunosuppressive cells, such as regulatory T cells (Tregs) and myeloid-derived suppressor cells (MDSCs), is often elevated in the TME of HCC, contributing to an immunosuppressive environment that facilitates tumor growth and metastasis (36). This immunosuppressive landscape can hinder the effectiveness of immune checkpoint inhibitors, which are increasingly used in HCC treatment. The interplay between copper homeostasis and the immune response is also critical. Copper is an essential trace element that modulates various immune functions, including the activation and differentiation of immune cells. Dysregulation of copper levels can lead to altered immune responses, potentially promoting tumorigenesis (37). For example, copper deficiency has been shown to impair the function of innate immune cells, while excess copper can enhance the immunosuppressive capabilities of certain immune cell populations (26). This dual role of copper underscores the complexity of its involvement in HCC and the immune microenvironment. To summarize, cuproptosis serves as a crucial mechanism of cellular demise, closely associated with the immune microenvironment in HCC. The modulation of copper concentrations alongside the expression of genes associated with cuproptosis can affect the infiltration of immune cells and shape the tumor’s overall immune environment. Gaining a deeper understanding of these relationships could yield important insights for the creation of targeted therapies and enhance the effectiveness of immunotherapy for patients with HCC. Additional investigations are required to clarify the specific mechanisms through which cuproptosis influences the immune microenvironment and to identify possible therapeutic approaches that utilize this information to achieve improved clinical results in HCC.

### 3.4 Cuproptosis and treatment resistance in HCC

Elevated copper levels have been associated with various malignancies, including HCC, where they contribute to tumor progression and treatment resistance. One of the key mechanisms by which copper influences treatment resistance is through its effect on cellular signaling pathways. For instance, copper is known to interact with several proteins involved in cell proliferation and survival. In HCC, high levels of copper can promote the expression of oncogenes, such as MYC, which is linked to increased tumor cell proliferation and invasiveness. This interaction suggests that copper may enhance the malignant characteristics of HCC cells, making them more resistant to therapeutic agents (38). Moreover, copper is implicated in the regulation of autophagy, a process that can contribute to drug resistance. Studies have shown that copper can modulate the autophagic response in HCC cells, which may help these cells survive under stress conditions, such as those induced by chemotherapy. For example, the structural maintenance of chromosome 4 (SMC4) has been identified as a potential marker of poor response to chemotherapy in HCC, and it may promote autophagy, thereby increasing drug resistance (39) (40). The role of copper in the context of specific chemotherapeutic agents is also significant. For instance, in the case of cisplatin, a commonly used chemotherapy drug for HCC, higher copper levels have been associated with increased resistance. Research indicates that the TR4 nuclear receptor can enhance the sensitivity of HCC cells to cisplatin by modulating the expression of ATF3, a transcription factor involved in stress responses. When TR4 is knocked down, HCC cells exhibit increased resistance to cisplatin, highlighting the importance of copper in mediating this effect (41) (42). Additionally, the phenomenon of cuproptosis, a newly identified form of cell death induced by copper, has emerged as a critical area of research. Cuproptosis is distinct from other forms of cell death, such as apoptosis and necroptosis, and is characterized by copper-induced proteotoxic stress. This process can lead to cell death in cancer cells, but the dysregulation of copper homeostasis can also contribute to resistance against therapies that aim to induce cell death (43). The relationship between copper levels and the effectiveness of chemotherapeutic agents like oxaliplatin has been explored, suggesting that targeting copper metabolism could enhance the efficacy of these treatments (44). Furthermore, the interaction between copper and lncRNAs has been identified as another layer of complexity in treatment resistance. LncRNAs can regulate various cellular processes, including drug resistance mechanisms. For example, the lncRNA NRAL has been shown to be involved in cisplatin resistance in HCC by regulating the Nrf2 signaling pathway, which is crucial for cellular defense against oxidative stress. This indicates that copper’s role in modulating lncRNA expression may also influence treatment outcomes (38).

## 4 Challenges and future directions for cuproptosis research

The specific molecular processes that govern cuproptosis remain inadequately defined. It is acknowledged that copper ions cause cell death by binding to fat acylated proteins involved in the tricarboxylic acid (TCA) cycle, which results in mitochondrial injury and proteotoxic stress; however, the precise pathways and interactions necessitate further exploration. This encompasses how cuproptosis correlates with other cell death modalities and the distinct roles played by crucial proteins such as FDX1 and lipoylated enzymes within this mechanism. Hepatocellular carcinoma (HCC) exhibits notable phenotypic and molecular diversity, complicating the examination of cuproptosis. Various HCC subtypes may exhibit differing responses to copper concentrations and interventions aimed at regulating copper levels. It is vital to identify and analyze these subtypes concerning cuproptosis to facilitate the creation of targeted treatment strategies. Although preclinical research has indicated a promising avenue in modulating copper homeostasis within HCC, applying these discoveries in clinical settings remains challenging. The advancement of effective therapies based on copper, including ionophores and chelators, should be informed by a deeper comprehension of individual patient factors such as genetic disparities and the interactions within the tumor microenvironment. The rise of resistance to standard therapies in HCC poses a major challenge. It is essential to comprehend how cuproptosis can be incorporated into current treatment strategies and how it may aid in overcoming this resistance. Further investigation is required to analyze the combined effects of copper-targeting agents with immunotherapy along with other therapeutic approaches. Future studies should aim to clarify the intricate mechanisms of cuproptosis, focusing on identifying downstream signaling pathways and understanding the role of ROS in facilitating cuproptosis. This could incorporate advanced methodologies such as single-cell RNA sequencing to examine cellular responses to copper in HCC. Developing innovative copper-based treatments that precisely target the distinct metabolic characteristics of various HCC subtypes could improve treatment effectiveness. This approach may include studying the use of nanoparticles for the targeted administration of copper ionophores or chelators to tumor locations. Exploring the possibilities of combining copper-targeting techniques with existing therapies, including immune checkpoint blockers or targeted treatments, could open new avenues for enhancing patient outcomes. Additionally, understanding how cuproptosis can influence the tumor immune microenvironment might unveil synergistic benefits. In summary, while the exploration of copper-induced death in liver cancer offers promising opportunities, tackling the challenges mentioned and pursuing future research directions will be vital for converting these insights into practical clinical applications. The potential of cuproptosis as a therapeutic focus in HCC could markedly reshape treatment strategies and enhance patient outcomes in the years to come.
